# Comparison The Effects of Two Monocyte Isolation Methods, Plastic Adherence and Magnetic Activated Cell Sorting Methods, on Phagocytic Activity of Generated Dendritic Cells

**Published:** 2013-08-24

**Authors:** Nowruz Delirezh, Ehsan Shojaeefar, Parva Parvin, Behnaz Asadi

**Affiliations:** Department of Microbiology, Faculty of Veterinary Medicine, Urmia University, Urmia, Iran

**Keywords:** Monocyte, Dendritic Cells, Phagocytosis, MACS, Adherence

## Abstract

**Objective:**

It is believed that monocyte isolation methods and maturation factors affect the phenotypic and functional characteristics of resultant dendritic cells (DC). In the present study, we compared two monocyte isolation methods, including plastic adherence-dendritic cells (Adh-DC) and magnetic activated cell sorting- dendritic cells (MACS-DC), and their effects on phagocytic activity of differentiated immature DCs (immDCs).

**Materials and Methods:**

: In this experimental study, immDCs were generated from plastic adherence and MACS isolated monocytes in the presence of granulocyte-macrophage colony-stimulating factor (GM-CSF) and interleukin 4 (IL-4) in five days. The phagocytic activity of immDCs was analyzed by fluorescein isothiocyanate (FITC)-conjugated latex bead using flow cytometry. One way ANOVA test was used for statistical analysis of differences among experimental groups, including Adh-DC and MACS-DC groups.

**Results:**

We found that phagocytic activity of Adh-DC was higher than MACS-DC, whereas the mean fluorescence intensity (MFI) of phagocytic cells was higher in MACS-DC (p<0.05).

**Conclusion:**

: We concluded that it would be important to consider phagocytosis parameters of generated DCs before making any decision about monocyte isolation methods to have fully functional DCs.

## Introduction

g wide variety of therapeutic approaches for cancer immunotherapy, the dendritic cell (DC) based vaccines have showed significant progression and successfully application against numerous types of tumors (1, 2) and some infectious agents such as human immunodeficiency virus (HIV) (3, 4). Also, in the context of cancer immunotherapy, these amazing cells can be used for specific cytotoxic T cell priming as passive immunotherapy or adoptive transfer (5). There are many reports of multiple methods for achieving substantial amounts of DCs, such as isolation from peripheral blood (6), culturing bone marrow cells in presence of granulocyte-macrophage colony-stimulating factor (GM-CSF) and interleukin-4 (IL-4) (7), differentiation from CD34^+^ cells in the presence of tumor necrosis factor-alpha (TNF-α) and GM-CSF (8), and peripheral blood monocyte using IL-4 and GM-CSF (9- 12). The later method has established the clinical phase of DC based immune therapy, and also, has been accepted as standard method to produce DC* in vitro* (13). Therefore, the monocyte isolation as a renewable source for DC generation have taken into account many of studies focusing on development of monocyte isolation methods, while their effects functional have been monitored on deriving DCs. Some of these methods are plastic/glass adherence (14), density gradient centrifugation (15, 16), as well as specific marker based separation such as magnetic activated cell sorting (MACS) (Miltenyi Biotec, Germany), fluorescent activated cell sorting, and bipolar tetrameric antibody (Ab) based separation (17), but the best one to be chosen is remained controversially. Indeed, above-mentioned methods may cause some changes to resultant DCs due to different composition of cells which were separated by each method. 

It is noted that the population of human monocytes are divided into two different subsets including CD14^low^CD16_+_ (5-10%) and CD14^+^ CD16^-^(90-95%) (16, 17); however, it has been demonstrated that the CD14^low^ CD16^+^ subset of monocytes Lake CD64 (18, 19) instead express lower level of CD32 (20) and higher level of CD11^c^ (21). It has been shown that the CD16 is in fact FcγRIII , which exhibits lowest affinity for its ligand compared to other counterparts, like CD32 (FcγRII) and CD64 (FcγRI), which show medium and highest affinity to their ligand, respectively (22). Furthermore, it is reported that CD11^c^ in combination with CD18, as a β2 integrin, enables the cells to adhere to plastic or glass (14, 23). Based on these findings, the composition of cells separated by these various methods may be different; in addition, FcγR mediated phagocytosis of these two subsets of monocyte is also varying with each other. Therefore, we expected phagocytosis parameters and antigen presentation capacity of resultant DCs to vary based on different isolation methods.

Herein, the phagocytic activity of generated DCs, as an important property affecting directly the antigen loading process, is investigated. In addition, immature DCs (immDcs) pick antigen up either by fluid-phase uptake (macropinocytosis) or by receptor-mediated internalization (endocytosis and phagocytosis). DCs express different receptors involving in antigen internalization, such as lectin type receptors (mannose receptor, CD205 and CD207), viral receptors (CD46), integrins and other receptors for apoptotic bodies (3â5á, 5â5á and CD36), complement receptors (CD35, CD88), as well as FcRs (FcγR, FcαR and FcεR binding to IgG, IgA, and IgE, respectively). These receptors lead to efficient antigen uptake and strongly enhance the efficiency of antigen presentation to T cells (24). In the present study, we compared the effects of two monocyte isolation methods, plastic adherence-dendritic cells (Adh-DC) and magnetic activated cell sorting-dendritic cells (MACS-DC), on phagocytic activity of generated DCs.

## Materials and Methods

### Media and reagents

In this experimental study, a complete tissue culture medium (CTM) including RPMI-1640 (Gibco, Germany) supplemented with 10% human AB serum (Blood Transfusion Organization, Tehran, Iran), 2.5×10^-5^ M 2ME, 2 mM L-glutamine (Sigma Chemical Co, Munich, Germany), 100 U/ml penicillin, and 100 µg/ml streptomycin (Sigma Chemical Co, Munich, Germany) were used to culture cells from peripheral blood mononuclear cells (PBMCs). Recombinant human GM-CSF (Novartis-Basel, Switzerland) and IL-4 (Peprotech-USA) were used to derive immDCs from peripheral blood monocytes. This research is confirmed by Ethics Committee of Urmia University, and informed consent was obtained from all participants.

### Monocyte isolation 

Fresh peripheral blood was taken from five healthy volunteers into sterile falcon tubes containing heparin (200 IU/ml) (Sigma Chemical Co., Munich, Germany). Peripheral blood mononuclear cells (PBMC) were isolated using Ficoll/Hypaque 1.077 g/ml (Sigma Chemical Co, Munich, Germany), as previously described (25).

ated from PBMC either by positive selection of CD14^+^ cells using a MACS system (Miltenyi Biotech, Bergisch Gladbach, Germany), according to the manufacturer’s protocol, or by cell culture flask adherence as plastic adherence method (referred to as Adh). For monocyte isolation by adherence, 10-15×10^6^ PBMC per flask were seeded into T25 cell culture flasks, and allowed to adhere in a 5% CO_2_ incubator at 37˚C for 2 hours in 5 ml of CTM. Non-adherent cells were removed and the adherent cells were carefully washed, twice with CTM. Monocytes isolated by MACS method were washed twice with CTM and seeded in T25 flask in the presence of GM-CSF and IL-4.

### Generation of immature DCs (immDCs)

For the generation of immDCs, monocytes isolated by either MACS or adherence were cultured in CTM supplemented with 800 U/ml human granulocyte-macrophage colony-stimulating factor (GMCSF) and 400 U/ml human IL-4 in a 5% CO_2_ , and 90% humidity at 37˚C for 5 days. After 3 days, the cells were fed again with the same doses of IL-4 and GM-CSF, then on day 4, apoptotic breast tumor cells, T47D cell line, irradiated by 8 Gy gamma radiation and incubated for 48 hours at 37˚C and 5% CO_2_ (5) (National Cell Bank, Pasteur Institute of Iran, Tehran, Iran) were added to immDCs at a ratio of 1:1. On day 5, immDCs were harvested and subjected to phagocytosis assays, throughout, DCs generated from monocytes obtained from MACS and Adherence methods were referred to as MACSDCs and Adh-DC, respectively.

### Phagocytosis test preparation

ImmDCs were subjected to phagocytosis assay on day 5. Afterwards, FITC-conjugated latex beads were opsonized by 10% human AB serum at concentration of 2.5×10^8^ beads/ml for 7.5 minutes at room temperature. It was followed by incubating immDCs and FITC-conjugated latex beads for 48 hours at 37˚C and 5% CO_2_ at the ratio of 1:20.

### Flow cytometry

The harvested cell were washed with quenching buffer (0.25% trypan blue and 13 µM citrate buffer in normal saline) three times (300×g for 10 minutes), and immDCs without beads were used as negative control. Phagocytic activity was analyzed in terms of percentage and mean fluorescence intensity (MFI) of positive cell on Dako flow cytometry system (Partec, Germany) and FlowMax software.

### Statistical analysis

The data depicted in each figure corresponds to one representative experiment of at least five independently performed experiments. One way ANOVA test was used for statistical analysis of differences among experimental groups. Difference at p value <0.05 was statistically considered significant. Data were expressed as mean ± standard deviation (SD), while two-tailed paired t test was used to determine the significance of data comparison.

## Results

**Fig 1:**
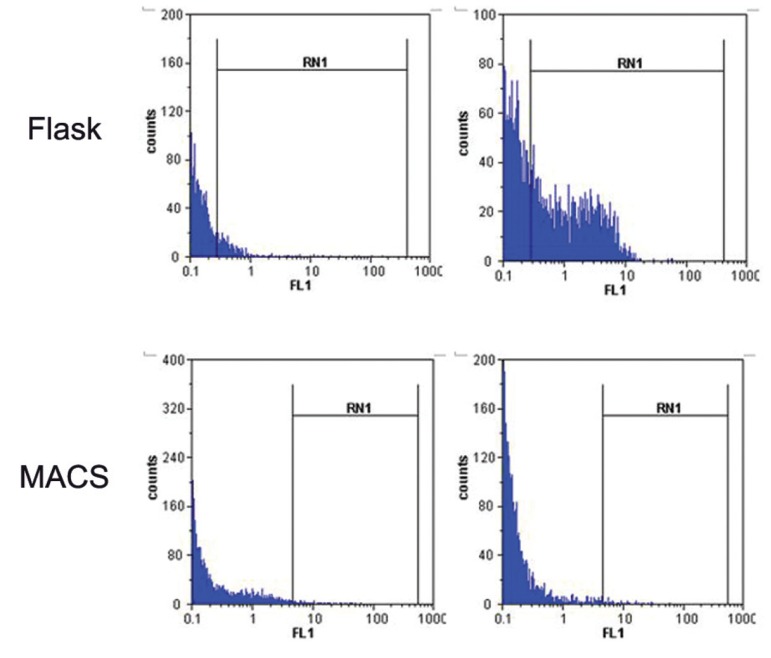
low cytomeric histograms obtained from phagocytic analysis of MACS-DC and Adh-DC. Both types of DCs were incubated with FITC- conjugated latex bead for 48 hours, then washed with quenching buffer and subjected to flow cytomeric analysis.

**Fig 2:**
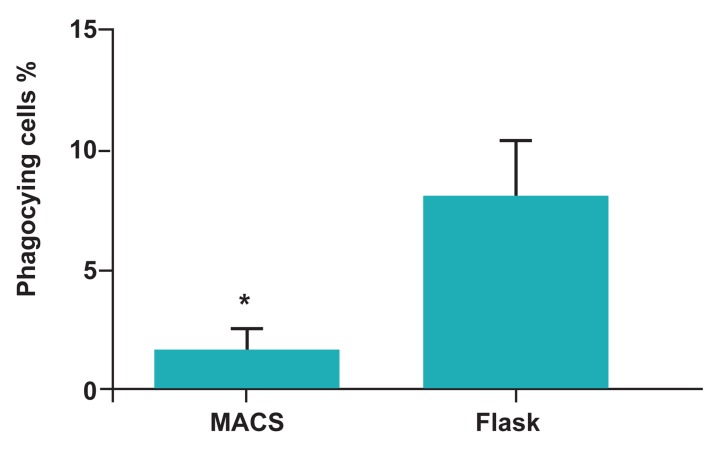
Flow cytometric analysis of phagocytic cells revealed significant decreased in number of phagocyting cells among MACS-DCs compared to Adh-DCs. Mean ± SD of five independent experiments. *; Represents significant difference between these two tested groups.

The viability of resultant DCs were 88.66 ± 8.08% and 89.66 ± 10.4% for adherence and MACS methods, respectively, whereas the yield of DCs were 5.69 ± 1.75% and 6.56 ± 2.49% of initial PBMC for adherence and MACS methods, respectively. Comparing the percentage of positive cells obtained from flow cytometric analysis, considered as percentage of phagocyting cells, revealed that Adh-DCs and MACS-DCs were performed phagocytosis 8 ± 2.35% and 1.51 ± 0.98%, respectively. These data showed the significant decreased percentage of phagocyting cells in MACS-DCs. (p<0.05; Figs 1, 2).

**Fig 3:**
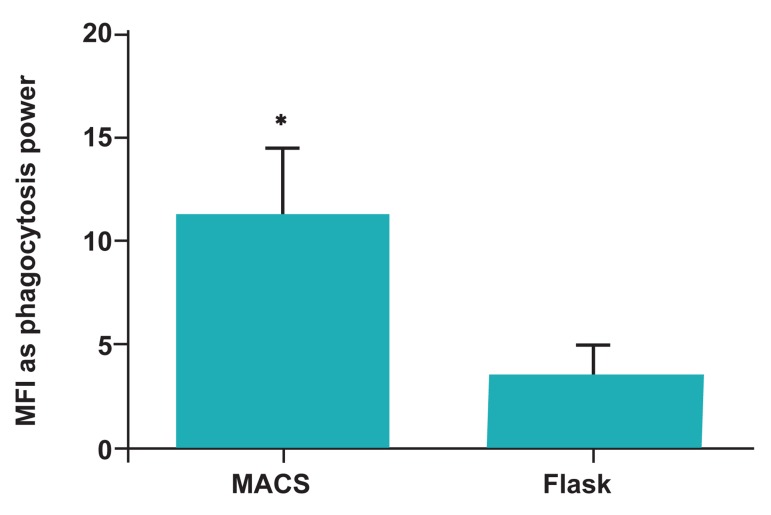
Flow cytometric analysis of phagocytic cells revealed significant increased MFI as phagocytosis power of MACSDCs in comparison to Adh-DCs. Mean ± SD of five independent experiments.

Another data obtained from Cell Quest software as MFI indicated that the relative phagocytosis power of MACS-DCs (11.09 ± 3.39) was higher than Adh-DCs (3.32 ± 1.63; p<0.05; Figs 1, 3).

**Fig 4:**
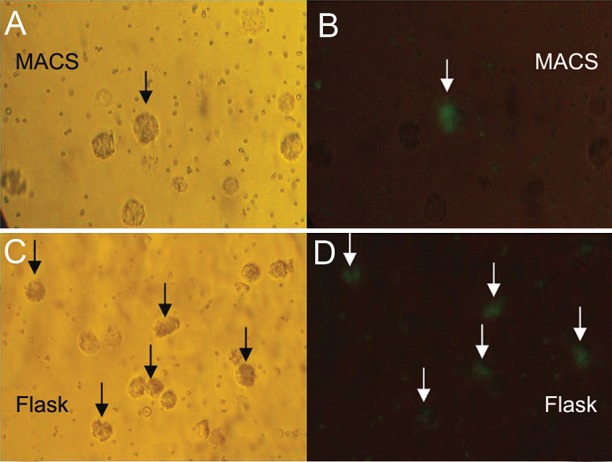
Microscopic view of phagocytic cells after 48 hours incubation (×200). MACS-DCs: visible light microscope view (A) fluorescent microscope view (B). Adh-DCs: visible light microscope view (C) fluorescent microscope view (D).

Also, the obtained data from flow cytometry could be matched with an image from microscopic examination (Fig 4).

*; Represents significant differ ence between these two tested groups.

## Discussion

Phagocytosis activity is an important property of immDCs which influences directly the* in vitro* tumor antigen loading. This process was followed by* in vitro* maturation used as a cell based vaccine or immunotherapy. As known, immDCs capture different type of antigens by multiple mechanisms and variety of receptors, and here, we discussed about the effect of MACS and adherence isolation methods of monocyte on resultant DCs.

As shown in results, the phagocyting cells were decreased in MACS method. Indeed, a few numbers of MACS-DCs performed phagocytosis regardless to their phagocytosis power. Whereas, evaluation of relative phagocytosis power of each cell, assessed by MFI, revealed a reversed result based upon truth of increased relative phagocytosis power of phagocyting MACS-DCs.

As mentioned above, the MACS technology uses the monocyte specific marker i.e. CD14, thus we can speculate that the majority (>90-95%) of obtained cell composition are CD14^+^ CD16^-^ monocytes due to lower expression of CD14 on CD14^low^CD16^+^ monocytes, leading to lower attraction at magnetic field. Also, the percentage of CD14^low^CD16^+^ subset in cell composition separated by plastic adherence can be higher than reported amount (5-10%), which is due to their further CD11^c^ expression causing more adhesion and chance of separation. Also, other β2 integrin expressing cells, such as natural killer cells and some lymphocytes (22), are subjected to be separated by adherence method.

If we accept this speculation, we can present some hypotheses which may justify our paradoxical results about phagocytosis.

Almost, all CD14^+^ CD16^-^ monocytes have filled their antigen capturing capacities using high affinity FcγRs (CD64 and CD32) for phagocytosis of apoptotic antigens which have been opsonized by existing AB serum before being harvested for phagocytosis assays. In contrast, the CD14lowCD16^+^ monocytes use low affinity FcγR (CD16) and low level of CD32, yet they all have not filled antigen capturing capacity.

The second hypothesis about losing of phagocytosis is indeed that most of magnetically separated monocytes might undergo early maturation and lose their phagocytosis activity according to physiological maturation process in which immDCs widely decrease their antigen uptake (26) and get the mature antigen presenting and T cell priming features (27, 28). On the other hands, there is a report in which magnetically separated monocytes were differentiated to mature DCs by a 48 hours culture protocol. In this report, 24 hours cultured monocytes in the presence of IL-4 and GM-CSF were considered as immDCs, giving rise to matured form by adding a cocktail of maturation factors in following 24 hours of culture (29). Relying on this report, we can conclude that our isolated monocytes either by MACS or by adherence differentiated to immDCs after 24 hours, so by engulfing, the added apoptotic antigens were transformed mostly to partially matured form on day 4, and thereby, majority of resultant cells lost their phagocytosis activity.

Collectively, in both groups, majority of cells did not phagocyte the fluorescent beads because the majority of their composition was CD14^+^ CD16^-^ monocytes with no antigen, capturing capacity or losing their phagocytosis power by maturation. But, the inscribed data of phagocytosis of fluorescent beads were attributable to the followings: i. A: CD14^low^CD16^+^ monocytes whose Ag capturing capacity was not filled or did not fully matured, while were constituted <5-10% and >5-10% of MACS-DCs and Adh-DCs population, respectively, and ii. B: Rare number of CD14^+^ CD16^-^ monocytes in both groups which did not phagocyte apoptotic antigen at all and remained intact.

Therefore, as shown in our results, the number of phagocyting cells in adherence group with more number of CD14^low^CD16^+^ monocytes could be higher; however, if the rare number of CD14^+^ CD16^-^ monocytes, enabling to phagocyte the fluorescent beads, were considered the same in both groups, the relative higher phagocytosis power of MACS-DCs could be reasonable because this subset performed phagocytosis were more powerful than CD14^low^CD16^+^ subset. For clarifying this phenomenon, read following example: If we consider that the phagocytosis power of CD14^+^ CD16^-^ monocyte is 20 and that of CD14^low^CD16^+^ monocyte is 10, we have 10 CD14^low^CD16^+^ and one CD14^+^ CD16^-^ monocytes in adherence group and one of both subset in MACS group, thus the average of 10×10 and 20 will be 10.9, whereas the average of 10 and 20 will be 15.

## Conclusion

According to these hypotheses, it can be concluded that monocyte isolation methods affect phagocytosis parameters of resultant DCs due to different composition of monocyte subsets by which being isolated. So, it would be important to decide which monocyte separation method must be used for achieving fully functional DCs. At the base of these results, we propose that the MACS method is better because DCs generated by this method loading more apoptotic antigen, and hereby, it is likely to get more maturation status. Also, its protocol is simple, and the isolated cells can be more homogenous. However, it should be noted that in the plastic adherence method, generated DCs are less manipulated and not exposed to magnetic field of MACS apparatus, so in term of clinical application of DC, one may prefer to use adherence method rather than MACS.
